# B7-H3 promotes the cell cycle-mediated chemoresistance of colorectal cancer cells by regulating CDC25A

**DOI:** 10.7150/jca.37255

**Published:** 2020-02-03

**Authors:** Yanchao Ma, Ruoqin Wang, Huimin Lu, Xiaomi Li, Guangbo Zhang, Fengqing Fu, Lei Cao, Shenghua Zhan, Zhenxin Wang, Zhongbin Deng, Tongguo Shi, Xueguang Zhang, Weichang Chen

**Affiliations:** 1 Department of Gastroenterology & Jiangsu Institute of Clinical Immunology, The First Affiliated Hospital of Soochow University, 188 Shizi Road, Suzhou, China.; 2 Jiangsu Key Laboratory of Clinical Immunology, Soochow University, 708 Renmin Road, Suzhou, China.; 3 Jiangsu Key Laboratory of Gastrointestinal tumor Immunology, The First Affiliated Hospital of Soochow University, 708 Renmin Road, Suzhou, China.; 4 James Graham Brown Cancer Center, Department of Microbiology &Immunology, University of Louisville, Kentucky 40202, USA.

**Keywords:** colorectal cancer, chemoresistance, B7-H3, CDC25A

## Abstract

Colorectal cancer (CRC) is one of the most common malignancies, and chemoresistance is one of the key obstacles in the clinical outcome. Here, we studied the function of B7-H3 in regulating cell cycle-mediated chemoresistance in CRC. The ability of B7-H3 in regulating chemoresistance was investigated via cell viability, clonogenicity, apoptosis and cycle analysis *in vitro*. Moreover, the role of B7-H3/CDC25A axis in regulating chemoresistance i*n vivo* in the xenograft tumor models by intraperitoneal injection of oxaliplatin (L-OHP) and CDC25A inhibitors. The correlation between B7-H3 and CDC25A was examined in the CRC patients by immunohistochemistry (IHC) and pathological analyses. We found that B7-H3 could effectively enhance the resistance to a chemotherapeutic drug (oxaliplatin or 5-fluorouracil) via CDC25A. B7-H3 regulated the expression of CDC25A by the STAT3 signaling pathway in CRC cells. Furthermore, overexpression of B7-H3 enhanced chemoresistance by reducing the G2/M phase arrest in a CDC25A-dependent manner. Silencing CDC25A or treatment with CDC25A inhibitor could reverse the B7-H3-induced chemoresistance of cancer cells. Moreover, both B7-H3 and CDC25A were significantly upregulated in CRC samples compared with normal adjacent tissues and that the levels correlated with tumor stage. CDC25A was positively correlated with B7-H3 expression in this cohort. Taken together, our findings provide an alternative mechanism by which CRC cells can acquire chemoresistance via the B7-H3/CDC25A axis.

## Introduction

With more than 600,000 deaths each year, colorectal cancer (CRC) has become the third most common cancer in the world according to the American Cancer Society in 2017 1. Chemotherapy is the optimal palliative therapy strategy for advanced CRC and is the first-line option for patients with metastatic CRC 2. However, a significant proportion of patients with CRC receiving chemotherapy become chemoresistant, with relapse and metastasis 3. Therefore, a better understanding of the molecular mechanisms associated with chemoresistance would help us to identify the subgroup of patients who may benefit from chemotherapy and avoid overtreatment.

Cell cycle-mediated chemoresistance is termed as a relative differential drug sensitivity based on the position of the tumor cells in the specific cell cycle phases 4. For example, both pharmacological and environmental factors arrested melanoma cells were in G1 phase to be resistant to bortezomib and temozolomide-mediated cytotoxicity 5. The leukemia stem cells with restricted cell cycle were characteristic of self-renewal, clonal evolution and therapeutic resistance 6. Si *et al.* showed that EZH2 silencing may reverse tamoxifen resistance in MCF-7 breast cancer cell by regulating the cell cycle 7. In lung cancer, the modification of cell cycle associated proteins was enhanced in cisplatin resistant A549 cells, which resulted in G2/M progression 8. Hence, these findings about cell cycle-mediated chemoresistance in cancers highlight that cell cycle status may alter the response of tumor cells to chemotherapic agents.

As an important immune checkpoint member of the B7-CD28 family, B7-H3 (B7 homology 3, CD276), is a type I transmembrane protein that plays a crucial role in the T cell-mediated immune response 9. Previous research has shown that B7-H3 is abundantly expressed in a number of cancer types, including lung, breast, prostate, kidney, pancreas, ovary, endometrium and colorectal cancer 10, 11. This elevated expression is often associated with a poor patient prognosis 11. In addition to its immunologic function, B7-H3 participates in a variety of cellular biological functions. These functions include cell growth, migration, invasion, epithelial to mesenchymal transition (EMT) and cancer stemness 12. This evidence suggests that B7-H3 may contribute to tumor initiation and the acquisition of tumor aggressiveness in a certain cellular microenvironment. In addition, B7-H3 affects the sensitivity to various anticancer drugs and targeted therapies in several cancer types, including CRC 13. Although some preliminary evidences indicated that B7-H3 could regulate the DNA repair mechanisms or cancer cell stemness to affect tumor cell chemoresistance 14, 15, many undefined mechanisms may be involved, and the effects of B7-H3 on the cell cycle-mediated chemoresistance of human CRC cells need to be thoroughly investigated.

In this study, we found that B7-H3 enhanced chemoresistance by reducing the G2/M phase arrest in a cell division cycle 25A (CDC25A)-dependent manner in CRC cells. Importantly, we demonstrated that CDC25A expression was critical for B7-H3-mediated CRC chemoresistance both *in vitro* and *in vivo*. Moreover, both B7-H3 and CDC25A were significantly upregulated in CRC samples compared with the normal adjacent tissues and correlated with tumor stage. Herein, our findings provided an alternative mechanism by which CRC cells can acquire and regulate the resistance to chemotherapy.

## Materials and methods

### Cell lines and cell culture

Normal colorectal cell (NCM460) and five CRC cell lines (HCT116, HT29, SW480, SW620, and RKO) were purchased from the Chinese Academy of Science Cell Bank. Cells were cultured in DMEM medium and RPMI-1640 (HyClone, Logan, Utah, USA) containing 10% fetal bovine serum (FBS, Gibco, Grand Island, New York, USA), 100 U/ml penicillin and 100 mg/ml streptomycin at 37 °C in a humidified atmosphere of 5% CO_2_.

### Cell lentivirus infection and transfection

Lentivirus vectors carrying human B7-H3 cDNA and B7-H3 shRNA were generated by Genechem Co. Ltd. (Shanghai, China). An empty backbone vector was used as a control. For lentivirus infection, HCT116 and RKO cells at 30% confluence in 6-well plates were transduced with lentiviral particles at a MOI of 20. The infection efficiency was confirmed by counting GFP-expressing cells under a fluorescence microscope 72 h after infection. Human CDC25A siRNA (5'-CAGGGAAUUUCAUUCCUCUTTAGAAGGAAUGAAAUUCCCUGTT-3') and its control siRNA (5'-UUCUCCGAACGAGUCACGUTTACGUGACACGUUCGGAGAATT-3') were purchased from GenePharma Co. Ltd. (Shanghai, China). HCT116 and RKO cells were transfected with siRNA reagents using Lipofectamine 2000 (Invitrogen, Carlsbad, CA, USA) according to the manufacturer's instructions. Transfection efficiency was determined by RT-qPCR and Western blot.

### RNA isolation and RT-qPCR

Total RNA from cultured cells was prepared using TRIzol reagent (TaKaRa, Shiga, Japan) according to the manufacturer's instructions. A total of 1 μg of RNA was reverse-transcribed using a cDNA Reverse Transcription Kit (TaKaRa) according to the manufacturer's instructions. The acquired cDNA was analyzed in triplicate by real-time PCR on CFX96 Touch^TM^ Real-Time PCR system (Bio-Rad, Hercules, CA, USA) using EvaGreen Dye (Biotium, Hayward, CA, USA). For the analysis of individual genes, 1 μg of total RNA was used for DNA synthesis, and RT-qPCR was conducted using a SYBR PrimeScript RT-qPCR Kit (Takara). The PCR conditions were as follows: 95 °C for 5 min, and then 40 cycles of amplification for 30 s at 95 °C, 45 s at 60 °C and 45 s at 72 °C. Individual gene expression was normalized to β-actin mRNA. The primer sequences for RT-qPCR are provided in Table S1.

### Protein extraction and Western blot analysis

NCM460 and CRC cells in 6-well plates were lysed with RIPA lysis buffer (ThermoScientific, Waltham, MA, USA) containing a protease inhibitor cocktail (Sigma, St. Louis, Missouri, USA) according to the manufacturer's instructions. Protein concentrations were measured with a Pierce BCA protein assay kit (ThermoScientific). Equal amounts of protein were separated by 10% SDS-PAGE and transferred to a PVDF membrane (Merck Millipore, Darmstadt, Germany). The antibodies use for Western blot analysis in this study were as follows: goat anti-human B7-H3 (R&D Systems, #AF1027), mouse anti-human STAT3 (CST, #9139), rabbit anti-human/mouse Phospho-STAT3 (CST, #9145), mouse anti-human CDC25A (Abcam, #ab2357), rabbit anti-human Cyclin B1 (CST, #12231), mouse anti-human CDK1 (CST, #9116), rabbit anti-human Bcl-2 (Abcam, #ab32124), rabbit anti-human/mouse Bax (Abcam, #ab32503) and mouse anti-human/mouse β-actin (CST, #3700). The membranes were developed with Clarity Western ECL substrate (Bio-Rad) and visualized with a ChemiDoc^TM^ MP imaging system (Bio-Rad).

### CCK8 assay

Cells were seeded in 96-well plates at an initial density of 5*10^3^ cells per well and incubated with oxaliplatin (L-OHP) or 5-Fluorouracil (5-FU) (Sigma) for 48 h. Then, the cells were stained with 10 μl of sterile CCK8 (Dojindo Laboratory, Japan) for 4 h at 37 °C. The absorbance at 450 nm was used as the reference wavelength. All experiments were conducted in quintuplicate.

### Lactate Dehydrogenase (LDH) release assay

The culture supernatant was collected from the CRC cells treated with or without 5 μM L-OHP or 10 μM 5-FU for 48 h. The LDH in the supernatants was tested using an LDH Cytotoxicity Assay Kit according to the manufacturer's instruction (Jiancheng Bioengineering LTD., Nanjing, China).

### Colony formation assays

Cells were plated in 12-well plates at 2*10^3^ cells per well and treated with 5 μM L-OHP for 2 h and then cultured for 10-14 d. The colonies were fixed with methanol for 30 min and then stained with 1% crystal violet (Sigma) for 30 min.

### Flow cytometry

Cells were harvested and washed with cold PBS, and the cell-cycle distribution and apoptosis rate were analyzed by flow cytometry (Beckman Coulter, CA, USA). Briefly, CRC cells were plated in 6-well plates at 10^5^ cells per well for 24 h. Then, the cells were treated with 0 μM, 20 μM or 40 μM L-OHP for 48 h. To analyze of cell-cycle distribution, cells were collected, washed twice with PBS, fixed in 70% ethanol containing 0.5% FBS and stored at -20 ℃ for at least 24 h. Samples were washed twice with PBS, treated with cell cycle assays (Fcmrcs, Nanjing, Jiangsu, China), and analyzed by flow cytometry using ModFit LT 3.1. For apoptosis analysis, the cells were washed with cold PBS and stained with Annexin V-PE and 7-AAD (BD, Franklin Lakes, NJ, USA), according to the manufacturer's instructions. Annexin-V+/7-AAD- cells and Annexin-V+/7-AAD+ cells were the apoptotic cells.

### Xenograft tumor model

Female nude mice (6-8 week, 18-20 g) were purchased from the Shanghai Laboratory Animal Center. All mice were synchronized with a 12 h light/dark cycle in an autonomous chronobiological animal facility (Suzhou, Jiangsu, China), with the lights on from 6 am (Zeitgeber time 0) to 6 pm (Zeitgeber time 12) for 1 week. All experimental procedures were approved by the Institutional Animal Care and Use Committee of Soochow University (Suzhou, China). Two *in vivo* experiments were designed. In experiment 1, the mice were divided randomly into the HCT116-EV (empty vector, EV), HCT116-B7-H3 (B7-H3), HCT116-EV+L-OHP (EV+L-OHP) and HCT116-B7-H3+L-OHP (B7-H3+L-OHP) groups (n=5 per group), and equal amounts of HCT116-B7-H3 or control cells (5*10^6^) were injected subcutaneously into the flank of each mouse. In experiment 2, the mice were randomly divided into HCT116-B7-H3+L-OHP (L-OHP), HCT116-B7-H3+L-OHP+Menadione (Menadione+L-OHP) and HCT116-B7-H3+L-OHP+DMSO (DMSO+L-OHP) groups (n=5 per group), and equal amounts of HCT116-B7-H3 (5*10^6^) were injected subcutaneously into the flank of each mouse. L-OHP was administered at a dose of 5 mg/kg at 10 am twice a week for 3 weeks. Menadione was given by oral administration (3 mg/kg). Treatment began on day 6, when the tumors were measurable. The tumors were examined every two days; the length and width measurements were obtained with calipers, and the tumor volumes were calculated. On day 21, the animals were euthanized, and the tumors were excised and weighed. Tumor size (mean ± SEM; mm^2^) was calculated according to the following equation: Tumor size (mm^2^) = S (mm) × L (mm), where S and L are the smallest and largest perpendicular tumor diameters, respectively 16.

### TUNEL assay

For the apoptosis assay, the xenografted tumor tissues of nude mice were determined using an *in situ* Cell Death Detection Kit (Roche Diagnostic, Mannheim, Germany) according to the manufacturer's instructions. Briefly, sections from paraffin-embedded tumor tissues were dewaxed and rehydrated, then incubated with TUNEL reaction mixture at 37 °C for 1 h in a chamber with humidified atmosphere. The nucleus was stained with DAPI. The numbers of TUNEL-positive cells and total cells were analyzed using a confocal microscope (Zessi, Jena, German).

### Patients and samples

From April 2010 to February 2014, 121 pairs of colorectal cancer tissue samples and the corresponding normal adjacent tissue samples were obtained from surgical procedures from the First Affiliated Hospital of Soochow University (Suzhou, China) with the consent of all patients. This study was approved by the Ethical Committee of Soochow University. The clinical pathological characteristics, including age, gender and TNM stage, were recorded (Table S2).

### Immunohistochemistry

Sections from paraffin-embedded tissues were incubated with a goat anti-human B7-H3 antibody (1:200, R&D Systems) or rabbit anti-human CDC25A antibody (1:100, Abcam) overnight at 4 °C. This step was followed by staining (45 min at room temperature) with the corresponding HRP-labeled rabbit anti-goat secondary antibody or goat anti-rabbit secondary antibody (Invitrogen). Next, the sections were visualized by staining with 3,3'-diaminobenzidine (Biocare Medical, California, USA) and counterstaining with hematoxylin (Sigma).

All sections were then reviewed blindly by two experienced pathologists (Dr. Cao and Dr. Zhan). The scoring criteria for B7-H3 and CDC25A immunostaining was using based on clinical data and adopted the semiquantitative immunoreactive score (IRS) system 17. Briefly, category A (intensity of immunostaining) was scored using the following criteria: 0, negative; 1, weak; 2, moderate; and 3, strong. Category B (percentage of immunoreactive cells) was scored using the following criteria: 1, (0-25%); 2, (26-50%); 3, (51-75%); and 4, (76-100%). Final scores were calculated by multiplying the scores of categories A and B in the same section; the scores ranged from 0 to 12.

### Statistical analysis

All statistical analyses were performed using GraphPad 6.0 statistical software packages. Statistically significant differences between groups were determined using Student's *t* test. A *P* value of <0.05 was considered statistically significant in all cases.

## Results

### B7-H3 imparts CRC cell chemoresistance

**Figure 1:**
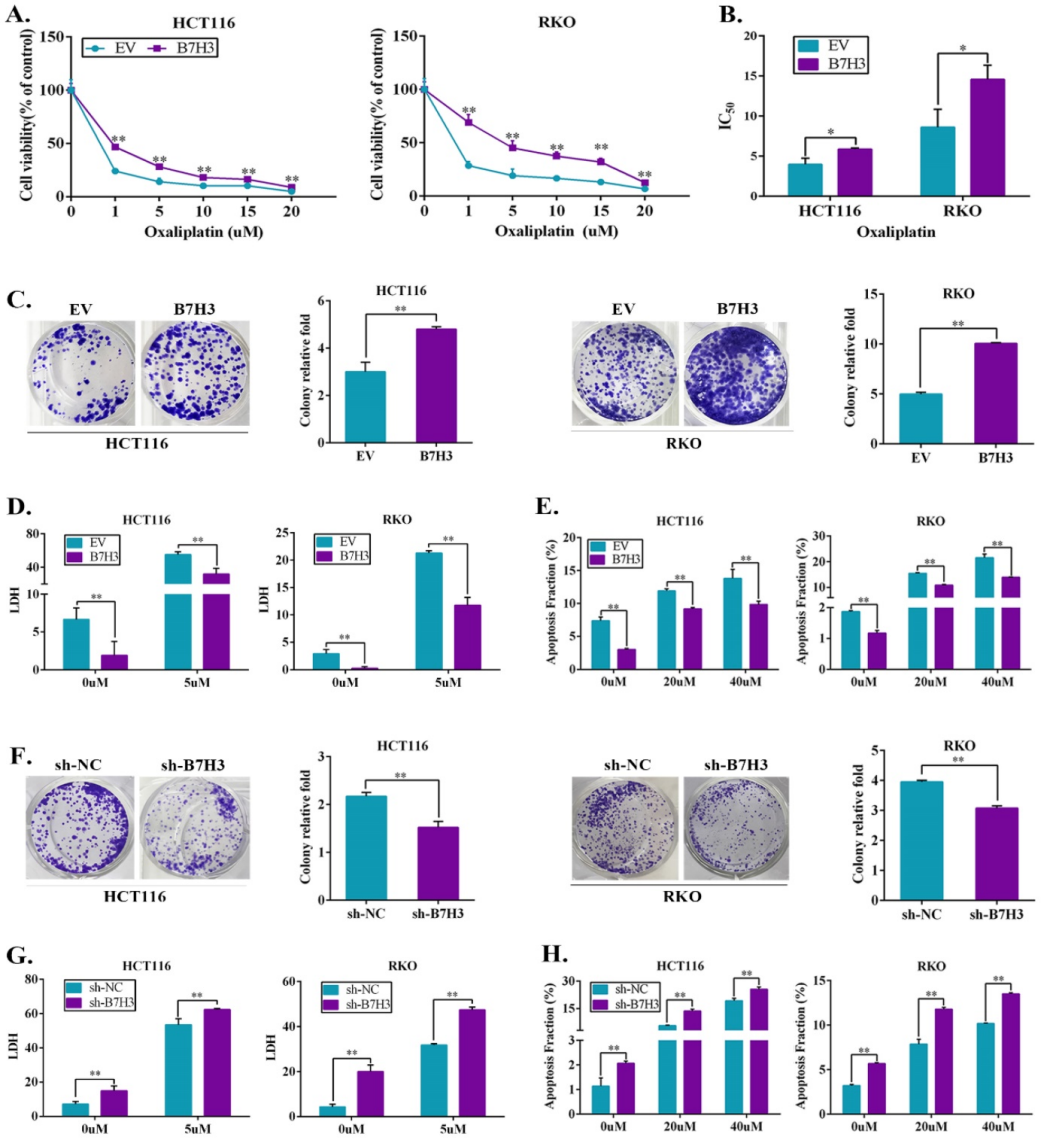
**Ectopic expression of B7-H3 promotes chemotherapy resistance. (A)** Cell viability after 48 h of L-OHP treatment was assessed by CCK8 in B7-H3-overexpressing HCT116 and RKO cells. Viability is relative to negative control cells as the mean ± SD. (n=5).** (B)** IC50 values were calculated on the basis of experiments from **A** as well as from negative control cells. Shown as the mean IC50 ± SD. (n=5). **(C)** The effect of B7-H3 overexpression on colony formation assay in HCT116 and RKO cells treated with 5 μM L-OHP for 2 h. **(D)** B7-H3-overexpressed HCT116 and RKO together with control cells were treated for 48 h with 5 μM L-OHP. Cell death was determined with the LDH assay. (n=5).** (E)** Apoptosis was measured using Annexin V/7-AAD double staining in B7-H3-CRC cells. **(F)** The effect of sh-B7-H3 on the colony formation assay in CRC cells treated with 5 μM L-OHP for 2 h.** (G)** B7-H3 knockdown HCT116 and RKO together with control cells were treated for 48 h with 5 μM L-OHP. Cell death was determined with the LDH assay. (n=5).** (H)** Apoptosis was measured using Annexin V/7-AAD double staining in sh-B7-H3-CRC cells. **P<0.01, *P<0.05.

As shown in Figure S1A, B7-H3 was frequently upregulated in the CRC cell lines (RKO, HCT116, HT29, SW480, and SW620) compared to the human colon healthy cell line (NCM460), suggesting B7-H3 overexpression has a crucial role in CRC progression. To examine whether B7-H3 expression has an impact on the chemoresistance of colorectal cancer cells *in vitro*, stable B7-H3-overexpressing HCT116 and RKO cell lines were established using a lentiviral delivery system. RT-qPCR and Western blot confirmed that both the protein and mRNA levels of B7-H3 were significantly upregulated in the B7-H3-overexpressing cell lines (B7-H3-HCT116 and B7-H3-RKO cells) (Figure S1B). Both B7-H3-overexpressing and control cells were incubated with different concentrations of oxaliplatin (L-OHP) or 5-fluorouracil (5-FU) for 48 h, and the viability of human CRC cells was assessed. The CCK8 assay results showed that L-OHP or 5-FU reduced cell viability in both B7-H3-overexpressing cells and control cells in a dose-dependent manner. However, B7-H3 overexpressing cells were more resistant to L-OHP and 5-FU than that of the control cells (Figure 1A and S1C). B7-H3 overexpression significantly increased the half maximum inhibitory concentration IC50 (causing 50% inhibition of viability) of L-OHP and 5-FU in HCT116 and RKO cells (Figure 1B and S1D). The colony formation assay showed that the number of colonies of B7-H3-HCT116 and B7-H3-RKO cells was increased compared to that of the control cells (Figure 1C). As shown in Figure 1D and Figure S1E, the results of LDH assay showed that B7-H3 overexpression suppressed the death of cells after L-OHP or 5-FU treatment. The flow cytometry-based apoptosis assay results showed that B7-H3 overexpression decreased the apoptotic populations in both CRC cell lines (Figure 1E and S1F). In addition, L-OHP treatment reduced Bcl-2 and induced Bax protein expression. The overexpression of B7-H3 effectively augmented Bcl-2 expression and suppressed Bax protein expression (Figure S1G).

We next examined the biological significance of B7-H3 in CRC cell chemoresistance by complementary loss-of-function studies. Stable B7-H3 knockdown HCT116 and RKO cell lines were established, and both the protein and mRNA levels of B7-H3 were significantly downregulated after knockdown (Figure S2A). As shown in Figure 1F, the clonogenic assay analysis revealed that knocking down B7-H3 significantly reduced the survival rate in HCT116 and RKO cell lines compared with the control cells (shRNA negative control, sh-NC). B7-H3 knockdown increased the LDH level of CRC cells after L-OHP or 5-FU treatment (Figure 1G and S2B). B7-H3 knockdown increased the apoptotic populations in HCT116 and RKO cells (Figure 1H and S2C). Consistently, the knockdown of B7-H3 further suppressed Bcl-2 protein expression and effectively augmented Bax expression in CRC cells (Figure S2D).

### B7-H3 reduces drug-induced G2/M phase arrest of CRC cells via upregulating CDC25A

**Figure 2:**
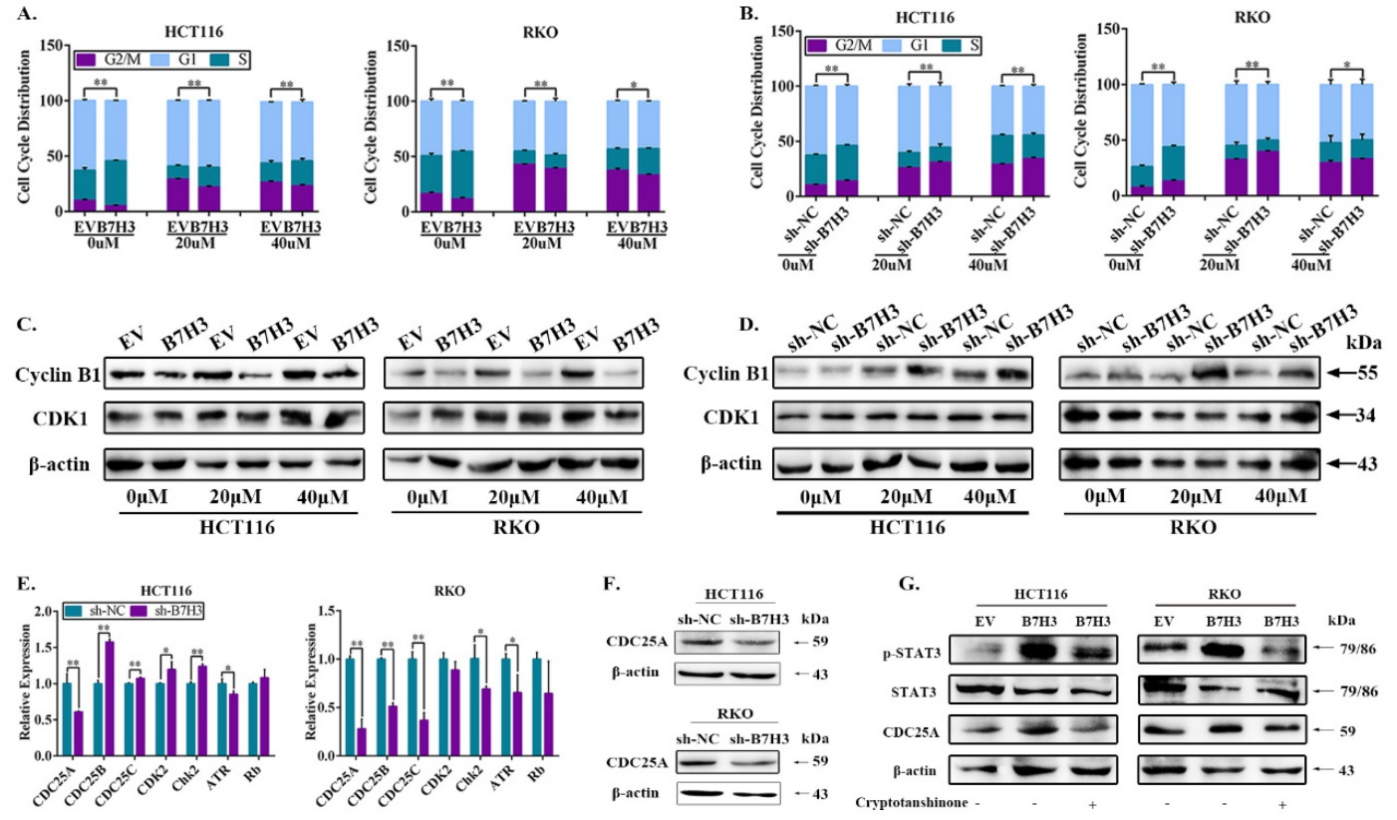
**B7-H3 inhibits CRC cells G2/M phase arrest via regulating CDC25A. (A)** The effect of B7-H3 overexpression on cell cycle progression in HCT116 and RKO cells. Cells were treated with or without 20 or 40 μM L-OHP for 48 h. After 48 h, both attached and floating cells were harvested for cell cycle analysis. **(B)** The effect of B7-H3 knockdown on cell cycle progression in HCT116 and RKO cells. Cells were treated with or without 20 or 40 μM L-OHP for 48 h. After 48 h, both attached and floating cells were harvested for cell cycle analysis.** (C)** Western blot analysis was used to analyze the protein levels of Cyclin B1 and CDK1 in control and B7-H3 overexpressed CRC cells with or without 20 or 40 μM L-OHP for 48 h. β-actin served as a loading control. **(D)** Western blot analysis of the protein levels of Cyclin B1 and CDK1 in control and B7-H3 knockdown CRC cells with or without 20 or 40 μM L-OHP for 48 h. β-actin served as a loading control. **(E)** RT-qPCR to determine the mRNA levels of CDC25A, CDC25B, CDC25C, CDK2, Chk2, ATR and Rb in both control and B7-H3 knockdown HCT116 and RKO cells. **(F)** Western blot analysis of the protein levels of CDC25A in control and B7-H3 knockdown CRC cells. β-actin served as a loading control. **(G)** Western blot analysis of the protein levels of STAT3, pSTAT3 and CDC25A in control and B7-H3 overexpression CRC cells with or without cryptotanshinone. β-actin served as a loading control. **P<0.01, *P<0.05.

Notably, drug-induced G2/M arrest can lead to apoptosis in various cancer cell lines, including CRC cells 18, we thus next determined whether B7-H3 modulated cell survival or apoptosis by reducing G2/M arrest of CRC cells under L-OHP treatment. Cell cycle analysis by flow cytometry was performed on CRC cells after treatment with 20 and 40 μM L-OHP. The B7-H3-overexpressing cells demonstrated a dramatically lower number of cells in the G2/M phase compared with the control cells (Figure 2A and S3A). Furthermore, the B7-H3 knockdown cells demonstrated a dramatically higher number of cells in the G2/M phase compared with the control cells (Figure 2B and S3B). In addition, we performed a Western blotting analysis to further investigate the effect of B7-H3 on the expression of G2/M phase-associated genes, such as Cyclin B1 and CDK1 (also named Cdc2) 19. B7-H3-overexpressing reduced, whereas B7-H3 knockdown induced, the expression of Cyclin B1 in CRC cells after treated with L-OHP (Figure 2C and 2D). However, B7-H3 had no effect on the CDK1 expression (Figure 2C and 2D). These results indicate that B7-H3 is able to regulate G2/M arrest in CRC exposed to L-OHP.

To further examine the accurate molecular mechanisms, we hypothesized that B7-H3 is involved in the G2/M checkpoint. Therefore, we assessed the expression of a spectrum of key G2/M checkpoint-related genes, including CDC25A, CDC25B, CDC25C, CDK2, Chk2, ATR and Rb in B7-H3 knockdown HCT116 and RKO cells by RT-qPCR. Compared with the control cells, the mRNA levels of CDC25A were obviously downregulated upon B7-H3 knockdown in both HCT116 and RKO cells (Figure 2E). Moreover, the mRNA levels of CDC25A were significantly upregulated in the B7-H3-overexpressing HCT116 and RKO cells (Figure S3C). In contrast, the knockdown of B7-H3 significantly reduced the protein levels of CDC25A in HCT116 and RKO cells (Figure 2F).

### B7-H3 regulates CDC25A expression via STAT3 pathway

To further comprehend the mechanism of B7-H3-mediated the expression of CDC25A, we reviewed works of literature. Barré B *et al.* has illustrated that STAT3 and its transcriptional cofactors were recruited to the promoter of CDC25A gene to activate its expression 20. In addition, our previous study showed that B7-H3 could promote aerobic glycolysis via STAT3/HK2 pathway 21. Hence, we hypothesized that the upregulation of CDC25A in both B7-H3-overexpressing CRC cells was STAT3 dependent. The activity of STAT3 significantly increased in both B7-H3-overexpressing HCT116 and RKO cells (Figure 2G). Moreover, we observed that cryptotanshinone, a STAT3 phosphorylation inhibitor, inhibited the phosphorylation of STAT3 and significantly decreased the expression of CDC25A in both B7-H3-overexpressing HCT116 and RKO cells (Figure 2G). These results suggested that B7-H3 could regulate the expression of CDC25A via the STAT3 signaling pathway in CRC cells.

### B7-H3 promotes chemoresistance of CRC cells via STAT3/CDC25A

**Figure 3:**
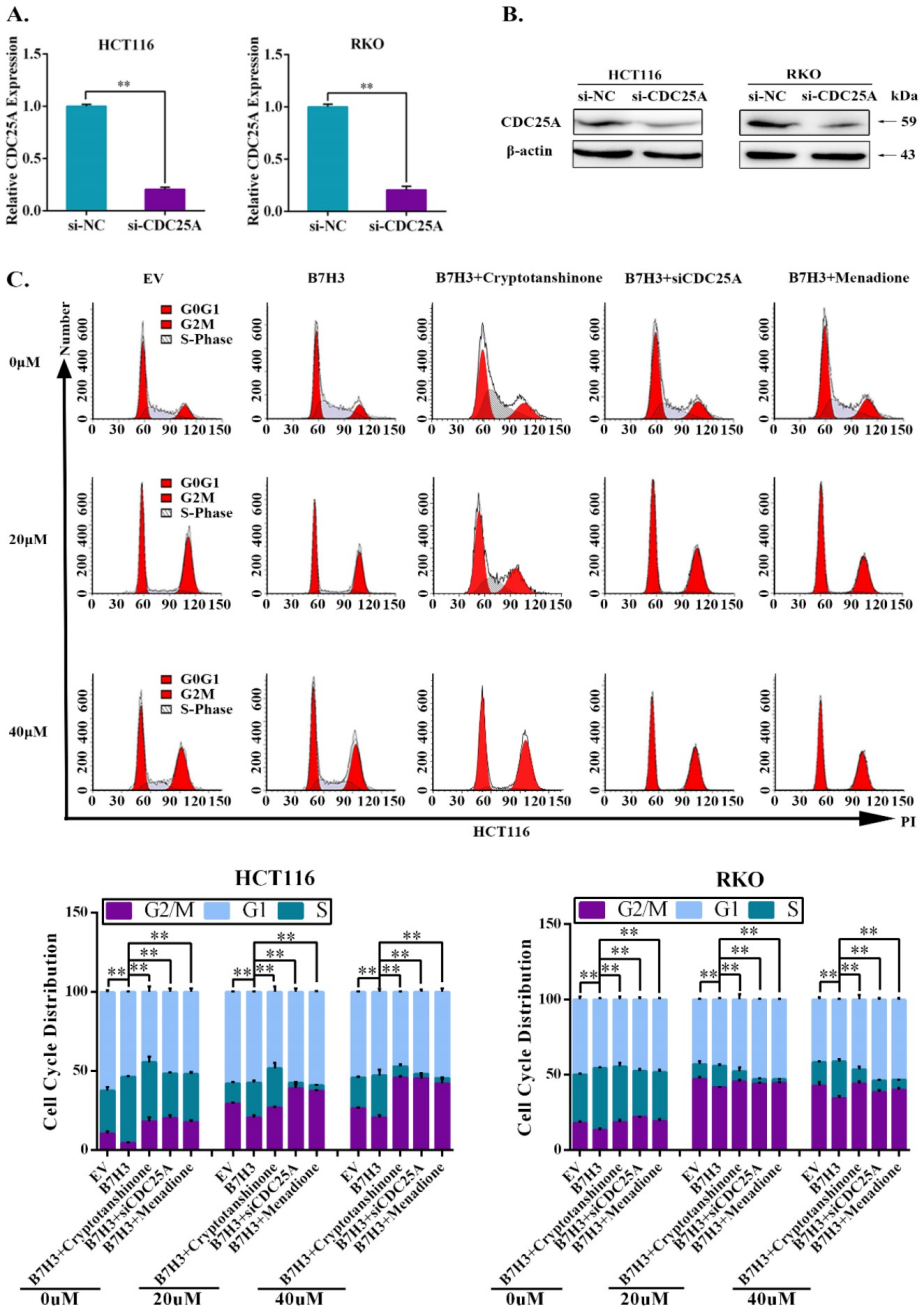
** STAT3/CDC25A increases G2/M arrest on B7H3 overexpression CRC cells. (A)** CDC25A mRNA in both HCT116 and RKO cells were analyzed by RT-qPCR after transfection with a siRNA negative control (NC) or CDC25A siRNA. **(B)** CDC25A protein level in both HCT116 and RKO cells were analyzed by Western blot after transfection with a siRNA negative control (NC) or CDC25A siRNA. β-actin served as a loading control.** (C)** The effect of STAT3/CDC25A silencing on cell cycle progression in control and B7-H3-overexpressing CRC cells. Cells were treated with or without 20 or 40 μM L-OHP for 48 h. After 48 h, both attached and floating cells were harvested for cell cycle analysis. **P<0.01, *P<0.05.

We further investigated the effect of STAT3/CDC25A on drug-induced G2/M phase arrest in B7-H3 overexpressing CRC cells. The expression of CDC25A decreased following the transfection with siRNA (Figure 3A and 3B). A cell cycle analysis showed that CDC25A silencing increased the percentage of CRC cells in the G2/M phase induced by B7-H3 overexpression (Figure 3C and S4). Additionally, Menadione, a CDC25A inhibitor, was used to treat B7-H3-overexpressing HCT116 and RKO cells. Menadione treatment also increased the percentage of CRC cells in the G2/M phase induced by B7-H3 overexpression (Figure 3C and S4). Furthermore, cryptotanshinone treatment increased the G2/M pahse arrest in B7-H3-overexpressed cells (Figure 3C and S4).

**Figure 4:**
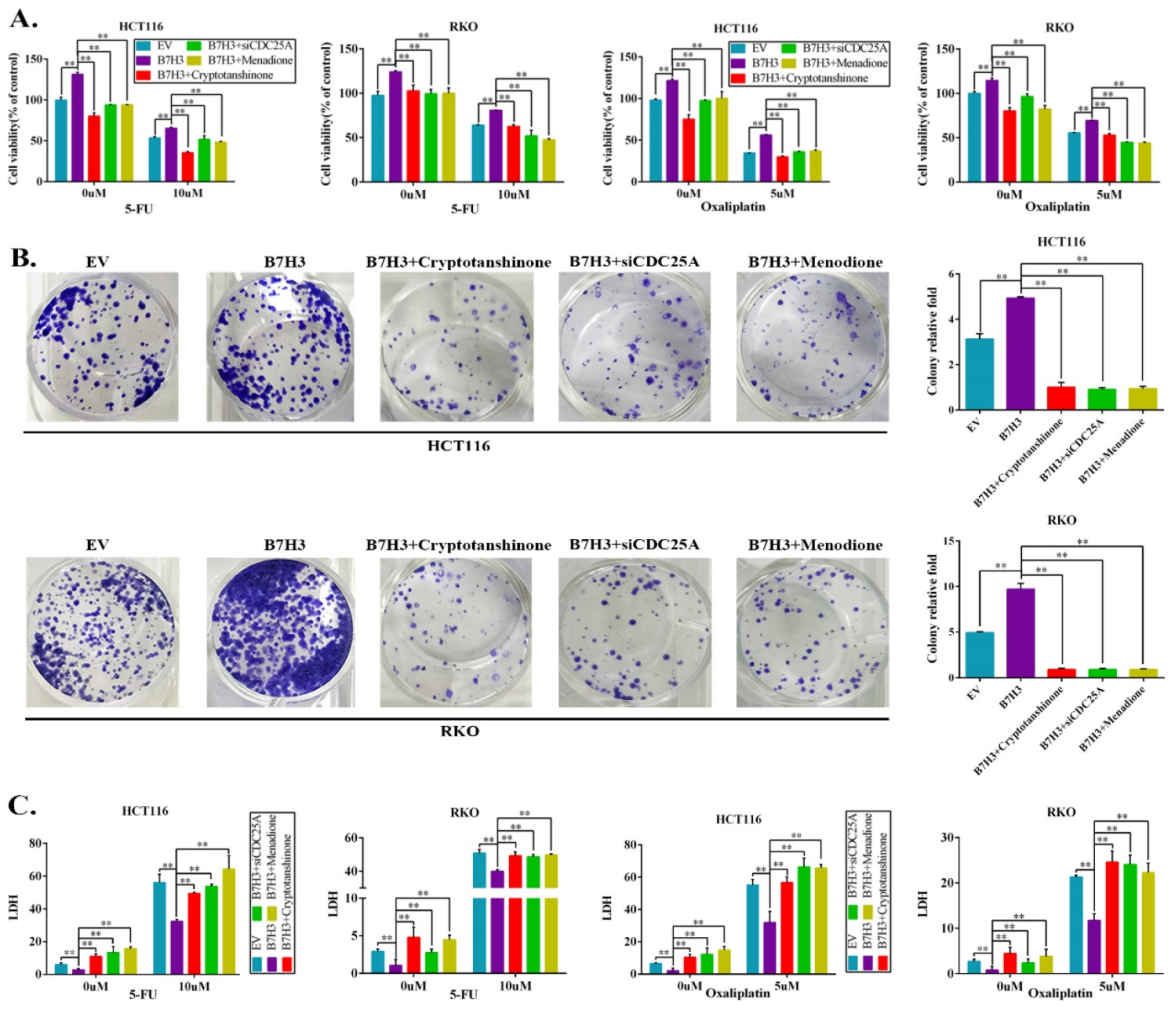
**B7-H3 promotes chemoresistance of CRC cells via STAT3/CDC25A. (A)** The CRC cell proliferation rates upon silencing STAT3/CDC25A in control or B7-H3-overexpressing CRC cells were determined using the CCK8 assay and are presented as the mean ± SD of three independent experiments. **(B)** The effect of silencing STAT3/CDC25A on the colony formation assay in control or B7-H3-overexpressing CRC cells treated with 5 μM L-OHP for 2 h. **(C)** The LDH level upon silencing STAT3/CDC25A in control and B7-H3-overexpressing CRC cells were determined with the LDH assay and are presented as the mean ± SD of three independent experiments. **P<0.01, *P<0.05.

The effects of the B7-H3/STAT3/CDC25A axis on the L-OHP resistance of CRC cell lines were examined. Cryptotanshinone treatment significantly moderated the cell survival rate, which was increased by B7-H3 overexpression in CRC cells after L-OHP or 5-FU treatment (Figure 4A). Silencing CDC25A or the inhibitor Menadione treatment produced consistent results (Figure 4A). In addition, both STAT3 inhibition and CDC25A suppression significantly reduced the colony formation efficiency, which was increased by B7-H3 overexpression in L-OHP-treated CRC cells (Figure 4B). Moreover, the LDH assay also confirmed the effects of B7-H3/STAT3/CDC25A axis on the L-OHP or 5-FU treatment of CRC cell lines (Figure 4C).

### The B7-H3/CDC25A axis confers CRC L-OHP resistance *in vivo*

**Figure 5:**
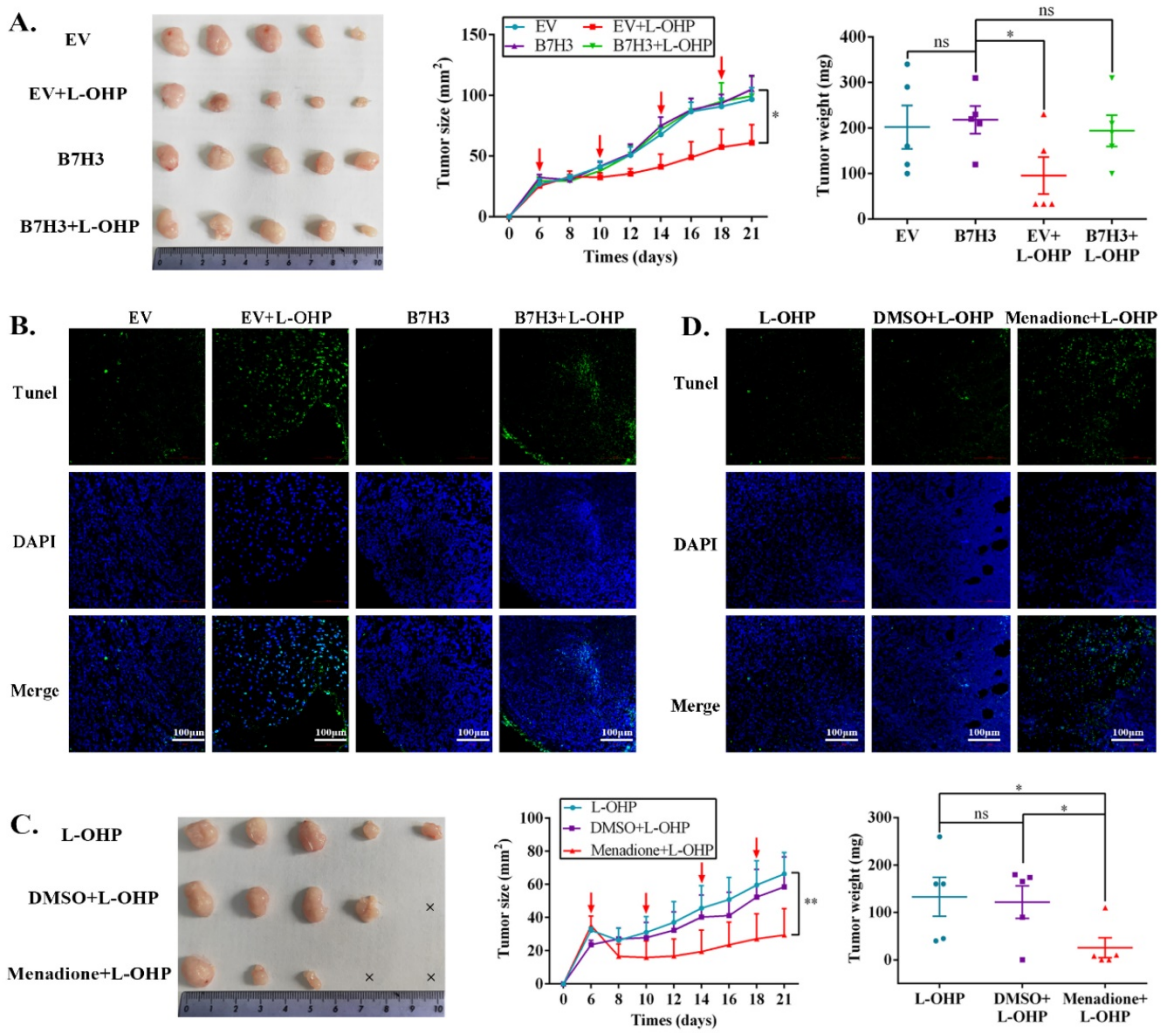
**The B7-H3/CDC25A axis confers L-OHP resistance *in vivo*. (A)** Representative images of tumors formed by EV-HCT116 or B7-H3-HCT116 with or without L-OHP treatment. The growth curves and weights of tumors formed by the indicated EV-HCT116 or B7-H3-HCT116 cells with or without L-OHP treatment. The data are presented as the mean ± SEM (n=5 mice per group).** (B)** TUNEL staining in tumor tissues of the nude mouse xenograft model with different treatments as indicated (scale bar, 100 μm) (right).** (C)** Representative images of tumors formed by B7-H3-HCT116+L-OHP with DMSO or Menadione treatment. The growth curves and weights of tumors formed by the indicated B7-H3-HCT116+L-OHP with DMSO or Menadione treatment. The data are presented as the mean ± SEM (n=5 mice per group). **(D)** TUNEL staining in tumor tissues of nude mice xenograft model with different treatments as indicated (scale bar, 100μm) (right). **P<0.01, *P<0.05.

To determine the effects of the B7-H3/CDC25A axis on CRC L-OHP resistance *in vivo*, xenograft models were produced. Nude mice bearing EV-HCT116 (empty vector, EV) or B7-H3-HCT116 xenografts were treated with L-OHP. Although the B7-H3-HCT116 xenograft tumors had no significant difference from the EV-HCT116 tumors in tumor size and weight, the B7-H3-HCT116 xenograft tumors grew faster than the EV-HCT116 tumors after L-OHP treatment (Figure 5A). Moreover, tumors that were derived from B7-H3-overexpressing HCT116 cells exhibited a considerable decrease in positive Terminal deoxynucleotidyl transferase dUTP nick-end labeling (TUNEL) staining compared with tumors derived from control vector HCT116 cells after L-OHP treatment (Figure 5B). We next confirmed whether B7-H3-regulated CRC chemoresistance was CDC25A-dependent *in vivo*. L-OHP and Menadione were administered to the B7-H3-HCT116 xenograft mice. We found that Menadione significantly reversed the resistance of B7-H3-HCT116 xenograft tumors to L-OHP *in vivo* (Figure 5C). Consistently, Menadione significantly increased the TUNEL-positive staining of B7-H3-HCT116 xenograft tumors after L-OHP treatment (Figure 5D). Taken together, these results showed that B7-H3 promotes CRC cell L-OHP resistance *in vivo* by inhibiting cell apoptosis in a CDC25A-dependent manner.

### Aberrant expression of B7-H3 and CDC25A are positively correlated in CRC patient tumor tissue specimens

**Figure 6:**
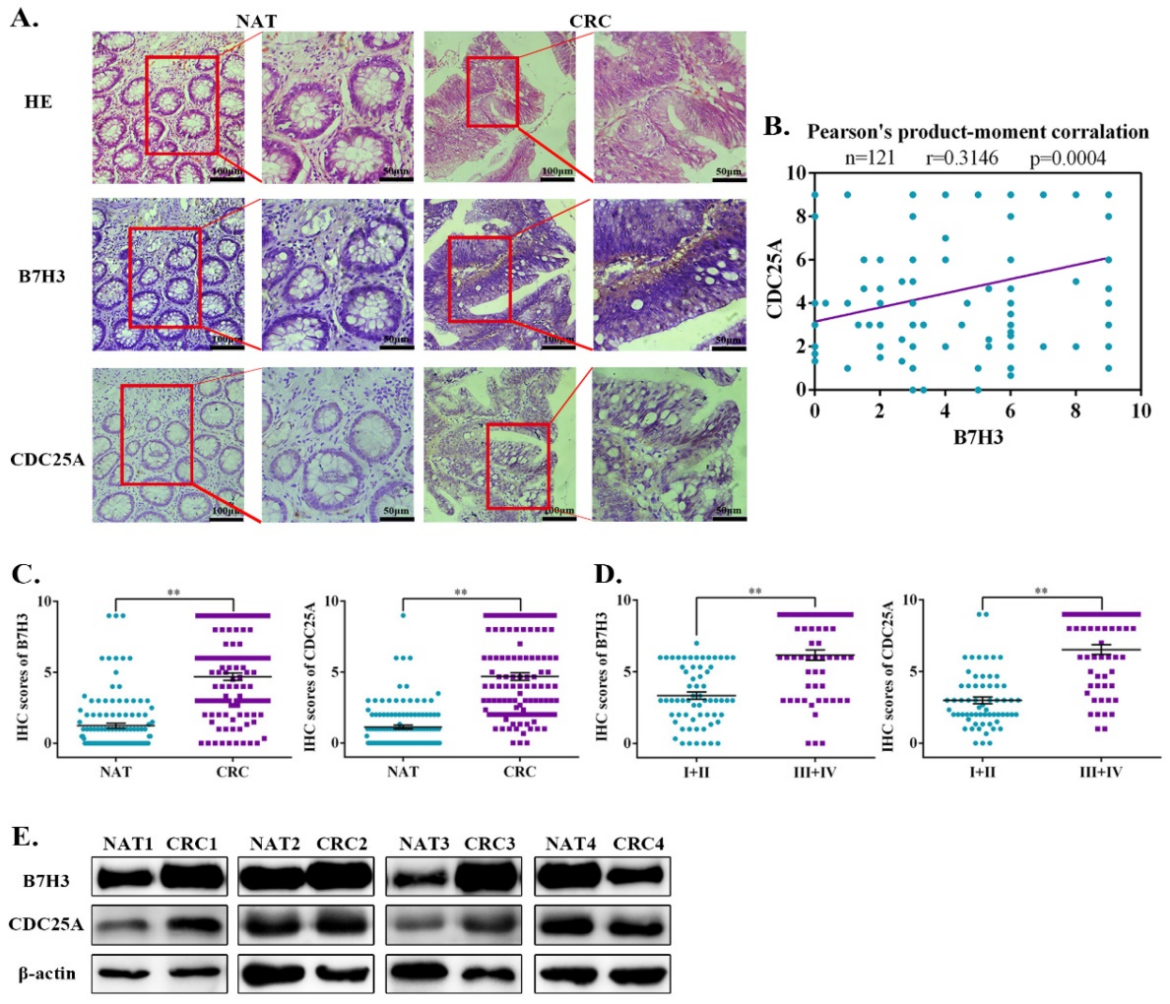
** Ectopic activation of the B7-H3/CDC25A axis indicates a poor prognosis in CRC patients. (A)** Images of IHC analysis of B7-H3 and CDC25A protein expression and hematoxylin and eosin (H&E) staining of CRC (n=121) tissue sections. B7-H3 and CDC25A protein expression based on their staining index in nonmalignant adjacent tissues (NAT) and CRC specimens. One representative image is shown. **(B)** Correlation analysis of the staining index of the expression levels of B7-H3 and CDC25A proteins in human CRC specimens (n=121). **(C, D)** B7-H3 **C** and CDC25A **D** protein expression based on their staining index in CRC specimens at different clinical stages. Values are expressed as the mean ± SEM. **(E)** B7-H3 and CDC25A protein levels in CRC and NAT cells were analyzed by Western blot. β-actin served as a loading control. **P<0.01, *P<0.05.

Previous studies documented that B7-H3 is aberrantly expressed in CRC and is consistently correlated with poor patient outcomes 22, 23. To investigate the clinical correlation between B7-H3 and CDC25A protein levels in CRC patient specimens, we analyzed 121 pairs of Chinese primary CRC lesions and the corresponding normal adjacent tissues. Immunohistochemistry staining results showed that both B7-H3 and CDC25A were significantly upregulated in CRC samples compared with the normal adjacent tissues (Figure 6A). Moreover, CDC25A was positively correlated with B7-H3 expression in this cohort (Figure 6B). Additionally, both B7-H3 and CDC25A expression increased with tumor stage. The levels of B7-H3 and CDC25A were higher in advanced clinical stages (III and IV) than those in early stages (I and II) (Figure 6C-6D, and Table S2). Furthermore, the Western blotting analysis revealed that B7-H3 and CDC25A protein expression was upregulated in 3 out 4 (75%) CRC samples (Figure 6E). These results indicated that B7-H3 and CDC25A are positively correlated in human CRC specimens.

## Discussion

Here, we reported that B7-H3-mediated resistance was associated with the ability of B7-H3 to inhibit drug-induced CRC cell apoptosis via regulating the G2/M phase in a CDC25A-dependent manner. B7-H3 promoted the expression of CDC25A via the STAT3 pathway. In addition, we demonstrated that both B7-H3 and CDC25A were significantly upregulated in CRC samples compared with the normal adjacent tissues and were associated with high-grade tumors. Moreover, CDC25A was positively correlated with B7-H3 expression in this cohort. Therefore, we conclude that B7-H3 may play a critical role in the regulation of cell cycle-mediated chemoresistance in CRC cells.

Numerous studies have supported that B7-H3 is intimately associated with chemotherapeutic resistance. B7-H3 promoted mantle cell lymphoma progression, and B7-H3 silencing enhanced the sensitivity of Maver and Z138 cells to rituximab and bendamustine 24. The downregulation of B7-H3 significantly decreased acute monocytic leukemia U937 cell growth and colony-forming ability and significantly enhanced the sensitivity of U937 cells to first-line chemotherapy drugs (idarubicin and cytarabine) 25. The silencing of B7-H3 was also observed to increase the sensitivity of the human pancreatic carcinoma cell line Patu8988 to gemcitabine as a result of enhanced drug-induced apoptosis 26. Furthermore, the silencing of B7-H3 increased the sensitivity of multiple human breast cancer cell lines to paclitaxel by abrogating Jak2/Stat3 phosphorylation 27. Ectopic expression of B7-H3 diminished the sensitization role of astragaloside IV, a component of Traditional Chinese Medicine Astragalus membranaceus, in cellular responses to cisplatin in non-small cell lung cancer 28. Moreover, B7-H3 could upregulate BRCC3 or XRCC1 expression to antagonize DNA damage caused by 5-FU or L-OHP in CRC 14, 29. In this study, we observed that the overexpression of B7-H3 promoted CRC cell colony formation and cell viability. Chemotherapy-induced apoptosis was significantly decreased in B7-H3-overexpressing CRC cells *in vitro* and *in vivo*. Meanwhile, sensitivity was dramatically increased in CRC cells with a stable knockdown of B7-H3. Therefore, the ectopic expression of B7-H3 promoted chemotherapy resistance.

Altered cell cycle regulatory genes can deregulate proliferation and cell cycle progression, which result in various pathophysiological conditions, including human malignancy 30, 31. In mantle cell lymphoma (MCL), miR-506 inhibited MCL cell proliferation, invasion and migration and caused cell cycle arrest at the G0/G1 phase by targeting B7-H3 32. In human non-small cell lung cancer, Physalin A could cause G2/M phase arrest and result in cell death of A549 cells 33. Zeng* et al*. reported that overexpression of the circadian clock gene Bmal1 could increase L-OHP sensitivity by regulating G2/M arrest 34. Our present results showed that B7-H3 overexpression decreased G2/M phase arrest, while B7-H3 knockdown increased G2/M phase arrest, which indicated that B7-H3 is involved in cell cycle-mediated chemoresistance via modulating cell cycle arrest.

STAT3, as a transcription factor, is identified as a positive oncogene participating in tumor advancement which activation inhibits apoptosis through the cell cycle, aerobic glycolysis, metastasis 16. Additionally, STAT3 also impacts the tumor microenvironment via stimulating angiogenesis and inflammation and via mediating immunosuppression 35, 36. Hence, STAT3 activation in tumor cells may promote tumor progression. In oesophageal carcinoma cells, STAT3 down-regulation could induce mitochondria-dependent G2/M cell cycle arrest and apoptosis 37. Liu *et al*. have illustrated that B7-H3 knockdown resulted in the reduction of STAT3 Tyr705 phosphorylation and increased paclitaxel sensitivity in breast cancer 27. A recent study showed that B7-H3 was involved in the activation of JAK2/STAT3 via redox-mediated oxidation and activation of Src in multiple myeloma cells 38. In CRC, B7-H3 augmented anti-apoptosis, invasion and migration of CRC cells through JAK2/STAT3 pathway 39-40. Similarly, our previous results indicated that B7-H3 overexpression activated the STAT3 pathway and promoted glycolysis in CRC 21. In this study, we found that B7-H3 contributed to STAT3 activity, as the inhibition of STAT3 using its inhibitory cryptotanshinone, could increase the arrest of the G2/M phase and LDH level, reduce cell viability in B7-H3 overexpressed CRC cells. Although both our results and other extensive research indicated that B7-H3 is able to activate the STAT3 pathway in multiple cancers, the precise way of B7H3 to regulate the STAT3 pathway is still unclear. Hence, we will further explore the way that B7-H3 regulates the STAT3 pathway in our next study.

Cell division cycle 25A (CDC25A), a member of the CDC25 phosphatase family, can inhibit the phosphorylation of the cyclin-dependent kinase (CDK) subunit, activate cyclin-CDK complexes and accordingly regulate cell cycle progression 41. Emerging evidence suggests that the expression of CDC25A is elevated in a number of human cancers and is often associated with high grade tumors and a poor prognosis 42. Moreover, cell cycle obstacles were accompanied by the overexpression of CDC25A in cancer tissues 43, indicating that CDC25A might regulate the cell cycle in the development and progression of tumors. It has been reported that CDC25A could assist both G1/S and G2/M progression in various types of cancers 43. Shen *et al.* reported that the novel phosphorylation of CDC25A (S76 and S82) induced by ciclopirox treatment was partially involved in the resistance to ciclopirox inhibition of cell proliferation 45. Additionally, Kajal *et al*. showed that BRE/BRCC45 facilitated the deubiquitylation of CDC25A by recruiting ubiquitin-specific-processing protease 7 (USP7) in the presence of DNA damage 46. In the present study, we found that the inhibition of CDC25A by Menadione or the knockdown of CDC25A expression effectively abolished B7-H3 overexpression-induced resistance to L-OHP and 5-FU *in vitro* and *in vivo*. Furthermore, previous study illustrated that pSTAT3 could bind to the promoter of CDC25A gene and activate its expression 20. In peripheral T-cell lymphomas, the reduction of STAT3 activity also decreased the expression of its target genes, such as MYC, PIM1, MCL1, CD30, IL2RA, CDC25A, IL4R 47. In this study, we found that B7-H3 could activate STAT3 and then upregulate CDC25A expression in CRC cells. Importantly, cryptotanshinone could inhibit the expression of CDC25A induction by B7-H3 in CRC cells. These data showed that B7-H3/STAT3 facilitated the G2/M transition via upregulating CDC25A in CRC, strongly suggesting that CDC25A is a vital bridge between B7-H3 and cell cycle-mediated chemoresistance.

Extensive DNA damage caused by chemotherapy drugs can induce cell apoptosis 48. Transactivation of CDC25A induced by NPAS2 can dephosphorylate Bcl-2(Thr69) and subsequently inhibit apoptosis in hepatocellular carcinoma 49. The expression of CDC25A was stabilized by NPAS2, which induced cell cycle progression and participated in the suppression of cell death by modulating caspase-3 cleavage, and the expression of Bcl2/Bax in acute myeloid leukemia 50. In addition, Wu *et al.* reported that the CHK1/CDC25A/CDK2 pathway is involved in HepG2 cell apoptosis in response to epirubicin by activating the p53/Bax/Caspase-3 pathway 51. We verified that B7-H3 could promote the anti-apoptotic protein Bcl-2 and inhibit the apoptotic protein Bax; the knockdown of B7-H3 increased cell apoptosis, indicating that the inhibition of cell apoptosis induced by the B7-H3/CDC25A axis may regulate Bcl-2 or Bax protein expression.

## Conclusion

To conclude, our study investigated the novel role of the B7-H3/STAT3/CDC25A axis in cell cycle-mediated chemoresistance of CRC. Disrupting the B7-H3/STAT3/CDC25A axis in CRC cells complicated chemotherapy-induced cell cycle arrest and apoptosis. Clinical analysis has indicated that the aberrant activation of the B7-H3/CDC25A axis is significantly related to tumor stage. Collectively, understanding the mechanism may help to provide a stricter prognosis and a more valid treatment for CRC patients with chemoresistance.

## Supplementary Material

Supplementary figures and tables.

## Abbreviations

CRC: Colorectal cancer
B7-H3: B7 homology 3, CD276
L-OHP: oxaliplatin
5-FU: 5-Fluorouracil
IC50: half maximal inhibitory concentration
CDC25A: Cell Division Cycle 25A
CDC25B: Cell Division Cycle 25B
CDC25C: Cell Division Cycle 25C
Chk2: Checkpoint kinase 2
ATR: ATM-Rad3-Related
Rb: Retinoblastoma gene
EV: empty vector
NC: negative control
