# B7-H3 promotes the cell cycle-mediated chemoresistance of colorectal cancer cells by regulating CDC25A: Erratum

**DOI:** 10.7150/jca.53041

**Published:** 2020-09-24

**Authors:** Yanchao Ma, Ruoqin Wang, Huimin Lu, Xiaomi Li, Guangbo Zhang, Fengqing Fu, Lei Cao, Shenghua Zhan, Zhenxin Wang, Zhongbin Deng, Tongguo Shi, Xueguang Zhang, Weichang Chen

**Affiliations:** 1 Department of Gastroenterology & Jiangsu Institute of Clinical Immunology, The First Affiliated Hospital of Soochow University, 188 Shizi Road, Suzhou, China.; 2 Jiangsu Key Laboratory of Clinical Immunology, Soochow University, 708 Renmin Road, Suzhou, China.; 3 Jiangsu Key Laboratory of Gastrointestinal tumor Immunology, The First Affiliated Hospital of Soochow University, 708 Renmin Road, Suzhou, China.; 4 James Graham Brown Cancer Center, Department of Microbiology &Immunology, University of Louisville, Kentucky 40202, USA.

In our paper 1, the western blot band for CDK1 in RKO cells (Figure 2D) was used wrong, when we put together Figure 2D. We apologize for the error and for any inconvenience that may cause to the readers and the editors of this journal. Figure 2D was collected as follows.

## Figures and Tables

**Figure 2:**
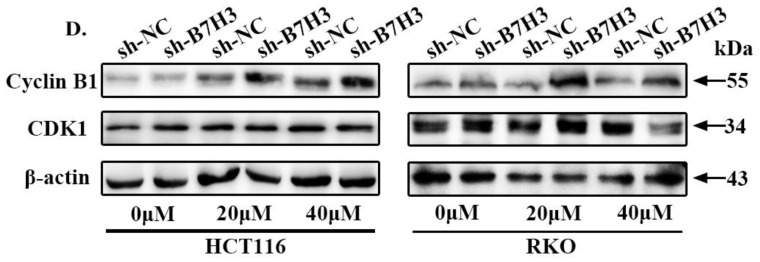
**B7-H3 inhibits CRC cells G2/M phase arrest via regulating CDC25A. (A)** The effect of B7-H3 overexpression on cell cycle progression in HCT116 and RKO cells. Cells were treated with or without 20 or 40 μM L-OHP for 48 h. After 48 h, both attached and floating cells were harvested for cell cycle analysis. **(B)** The effect of B7-H3 knockdown on cell cycle progression in HCT116 and RKO cells. Cells were treated with or without 20 or 40 μM L-OHP for 48 h. After 48 h, both attached and floating cells were harvested for cell cycle analysis.** (C)** Western blot analysis was used to analyze the protein levels of Cyclin B1 and CDK1 in control and B7-H3 overexpressed CRC cells with or without 20 or 40 μM L-OHP for 48 h. β-actin served as a loading control. **(D)** Western blot analysis of the protein levels of Cyclin B1 and CDK1 in control and B7-H3 knockdown CRC cells with or without 20 or 40 μM L-OHP for 48 h. β-actin served as a loading control. **(E)** RT-qPCR to determine the mRNA levels of CDC25A, CDC25B, CDC25C, CDK2, Chk2, ATR and Rb in both control and B7-H3 knockdown HCT116 and RKO cells. **(F)** Western blot analysis of the protein levels of CDC25A in control and B7-H3 knockdown CRC cells. β-actin served as a loading control. **(G)** Western blot analysis of the protein levels of STAT3, pSTAT3 and CDC25A in control and B7-H3 overexpression CRC cells with or without cryptotanshinone. β-actin served as a loading control. **P<0.01, *P<0.05.
