# Supplementary material for: B7-H3 promotes the cell cycle-mediated chemoresistance of colorectal cancer cells by regulating CDC25A
Source: J Cancer. 2020 Feb 3;11(8):2158–70. doi: 10.7150/jca.37255 (PMC7052923; doi:10.7150/jca.37255)
Supplement: Supplementary file 1 — Supplementary figures and tables. [file jcav11p2158s1.pdf]

**Supplementary data to:**

**B7-H3 promotes the cell cycle-mediated chemoresistance of colorectal cancer cells by regulating CDC25A**

**Table S1.** Primers for RT-qPCR assay of gene

| **Primer Name** | **Primer Sequence (5’-3’)** |
| --- | --- |
| Homo-B7-H3 Forward | ACAGGGCAGCCTATGACATT |
| Homo-B7-H3 Reverse | CTGCATTCTCCTCCTCACAG |
| Homo-CDC25A Forward | GTGAAGGCGCTATTTGGCG |
| Homo-CDC25A Reverse | TGGTTGCTCATAATCACTGCC |
| Homo-CDC25B Forward | ACGCACCTATCCCTGTCTC |
| Homo-CDC25B Reverse | CTGGAAGCGTCTGATGGCAA |
| Homo-CDC25C Forward | TCTACGGAACTCTTCTCATCCAC |
| Homo-CDC25C Reverse | TCCAGGAGCAGGTTTAACATTTT |
| Homo-CDK2 Forward | CCAGGAGTTACTTCTATGCCTGA |
| Homo-CDK2 Reverse | TTCATCCAGGGGAGGTACAAC |
| Homo-Chk2 Forward | CCCAAGGCTCCTCCTCACA |
| Homo-Chk2 Reverse | AGTGAGAGGACTGGCTGGAGTT |
| Homo-ATR Forward | GGCCAAAGGCAGTTGTATTGA |
| Homo-ATR Reverse | GTGAGTACCCCAAAAATAGCAGG |
| Homo-Rb Forward | ACTCTTCAGCAATTGGAAAGG |
| Homo-Rb Reverse | CACCAATGCAGAATTTATTTCAGT |
| Homo-β-actin Forward | CATGTACGTTGCTATCCAGGC |
| Homo-β-actin Reverse | CTCCTTAATGTCACGCACGAT |

**Table S2.** Clinical characteristics of patients

| CRC patients | Number | B7H3 expression P value | CDC25A expression P value |
|---|---|---|---|
| No. of patients | 121 | | |
| Gender | | | |
| Male | 71 | | |
| Female | 50 | 0.2468 | 0.5612 |
| Age (years) | | | |
| Mean | 60.42 | | |
| Range | 26-81 | 0.4016 | 0.5551 |
| Tumor location | | | |
| Colon | 70 | | |
| Rectum | 51 | 0.2165 | 0.2466 |
| TNM stage | | | |
| I-II | 63 | | |
| III-IV | 58 | < 0.0001 | < 0.0001 |

## Supplementary Figure Legends

**Figure S1. B7-H3 imparts CRC cell chemoresistance. (A)** B7-H3 protein levels in NCM460, RKO, HCT116, HT29, SW480 and SW620 cells were analyzed by Western blot. β-actin served as a loading control. **(B)** B7-H3 protein and mRNA levels in B7-H3-overexpressing HCT116 and RKO cells were analyzed by Western blot and RT-qPCR. β-actin served as a loading control. **(C)** Cell viability after 48 h of 5-FU treatment was assessed by CCK8 in B7-H3-overexpressing HCT116 and RKO cells. Shown relative to negative control cells as the mean ± SD. (n=5). **(D)** IC50 values were calculated on the basis of experiments from **C** as well as from negative control cells. Shown as the mean IC50 ± SD. (n=5). **(E)** B7-H3-overexpressed HCT116 and RKO together with control cells were treated for 48 h with 10 μM 5-FU. Cell death was determined with the LDH assay. (n=5). **(F)** Apoptosis was measured using Annexin V/7-AAD double staining in B7-H3-overexpressing CRC cells. These data are shown as the mean ± SD of three independent experiments. **(G)** Bcl-2 was upregulated while Bax was downregulated upon B7-H3 overexpression in both HCT116 and RKO cells compared with control cells.

**Figure S2. B7-H3 inhibits chemotherapy-induced apoptotic cell death of CRC cells. (A)** B7-H3 protein and mRNA levels in B7-H3 knockdown HCT116 and RKO cells were analyzed by Western blot and RT-qPCR. β-actin served as a loading control. **(B)** B7-H3-knockdown HCT116 and RKO together with control cells were treated for 48 h with 10 μM 5-FU. Cell death was determined with the LDH assay. (n=5). **(C)** Apoptosis was measured using Annexin V/7-AAD double staining in sh-B7-H3-CRC cells. These data are shown as the mean ± SD of three independent experiments. **(D)** Bax was upregulated while Bcl-2 was downregulated upon B7-H3 knockdown in both HCT116 and RKO cells compared with control cells.

**Figure S3. B7-H3 inhibits CRC cells G2/M phase arrest via regulating CDC25A. (A)** The effect of B7-H3 overexpression on cell cycle progression in HCT116 and RKO cells. Cells were treated with or without 20 or 40 μM L-OHP for 48 h. After 48 h, both attached and floating cells were harvested for cell cycle analysis. **(B)** The effect of B7-H3 knockdown on cell cycle progression in HCT116 and RKO cells. Cells were treated with or without 20 or 40 μM L-OHP for 48 h. After 48 h, both attached and floating cells were harvested for cell cycle analysis. **(C)** RT-qPCR to determine the mRNA levels of CDC25A in both control and B7-H3 overexpression HCT116 and RKO cells.

**Figure S4. STAT3/CDC25A increases G2/M arrest on B7-H3 overexpression CRC cells.** The effect of STAT3/CDC25A silencing on cell cycle progression in control and B7-H3-overexpressing CRC cells. Cells were treated with or without 20 or 40 μM L-OHP for 48 h. After 48 h, both attached and floating cells were harvested for cell cycle analysis.

## Figure S1

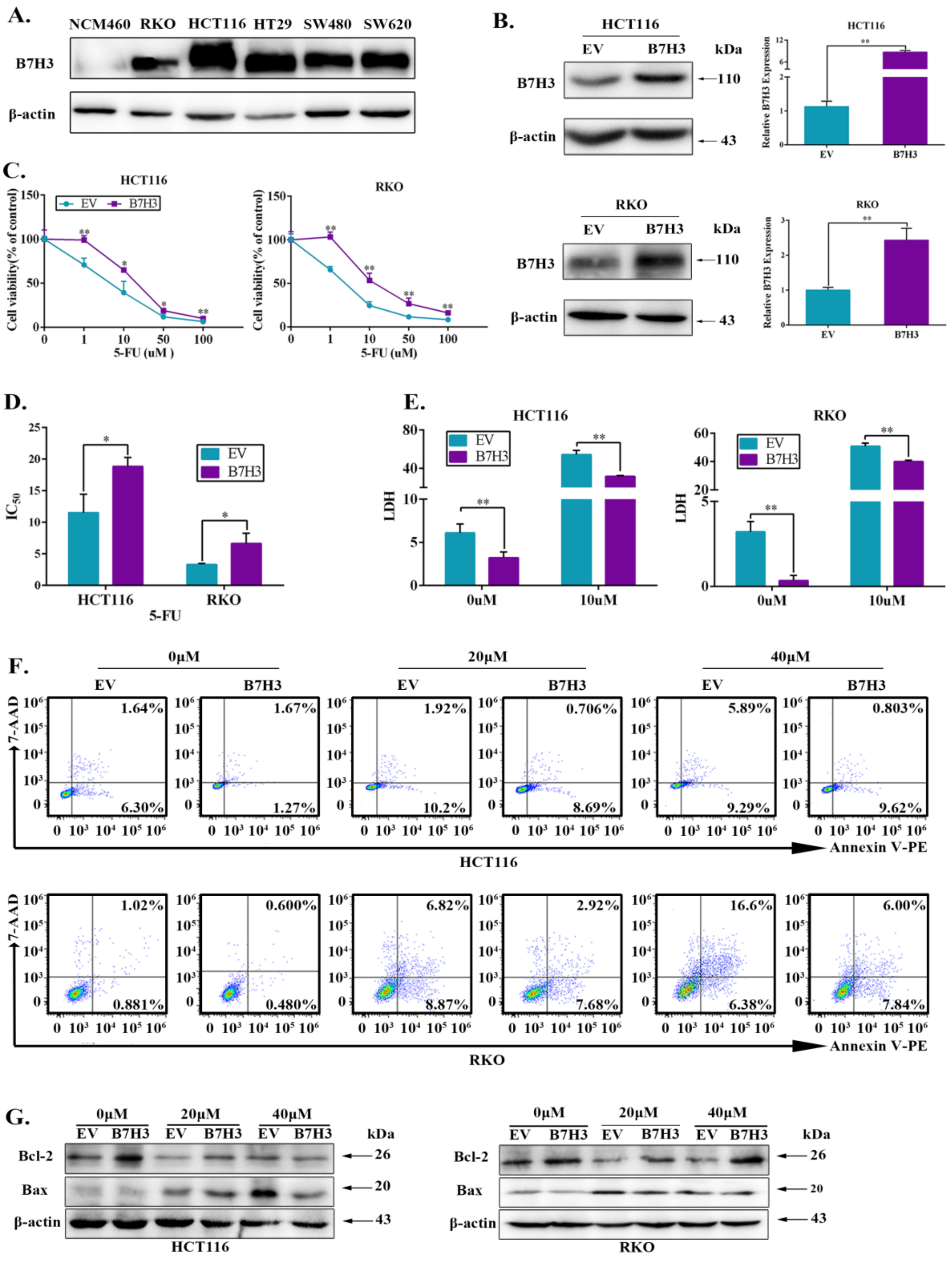
A.
NCM460 RKO HCT116 HT29 SW480 SW620
B7H3
β-actin
B.
HCT116
EV B7H3 kDa
B7H3
110
β-actin
43
Relative B7H3 Expression
EV
B7H3
RKO
C.
HCT116
RKO
Cell viability(% of control)
5-FU (uM )
5-FU (uM)
D.
IC50
HCT116
RKO
5-FU
E.
LDH
0uM
10uM
F.
0μM
20μM
40μM
7-AAD
Annexin V-PE
1.64%
6.30%
1.67%
1.27%
1.92%
10.2%
0.706%
8.69%
5.89%
9.29%
0.803%
9.62%
1.02%
0.881%
0.600%
0.480%
6.82%
8.87%
2.92%
7.68%
16.6%
6.38%
6.00%
7.84%
G.
Bcl-2
Bax
26
20

**Figure S2**

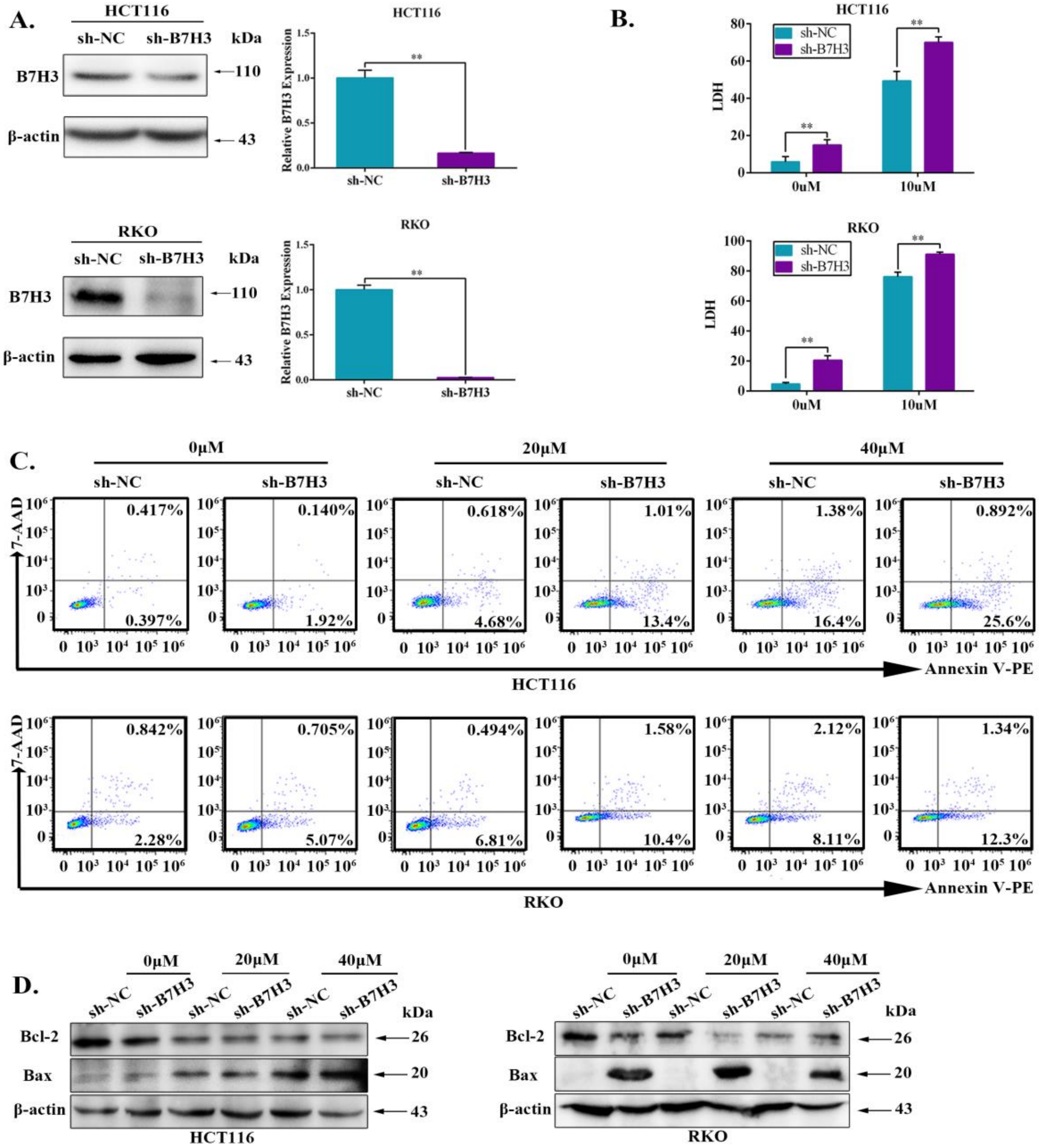

A.
HCT116
sh-NC
sh-B7H3
kDa
B7H3
110
β-actin
43
Relative B7H3 Expression
**
RKO
B.
LDH
sh-NC
sh-B7H3
0uM
10uM
C.
0μM
20μM
40μM
7-AAD
Annexin V-PE
0.417%
0.397%
0.140%
1.92%
0.618%
4.68%
1.01%
13.4%
1.38%
16.4%
0.892%
25.6%
0.842%
2.28%
0.705%
5.07%
0.494%
6.81%
1.58%
10.4%
2.12%
8.11%
1.34%
12.3%
D.
Bcl-2
26
Bax
20

**Figure S3**

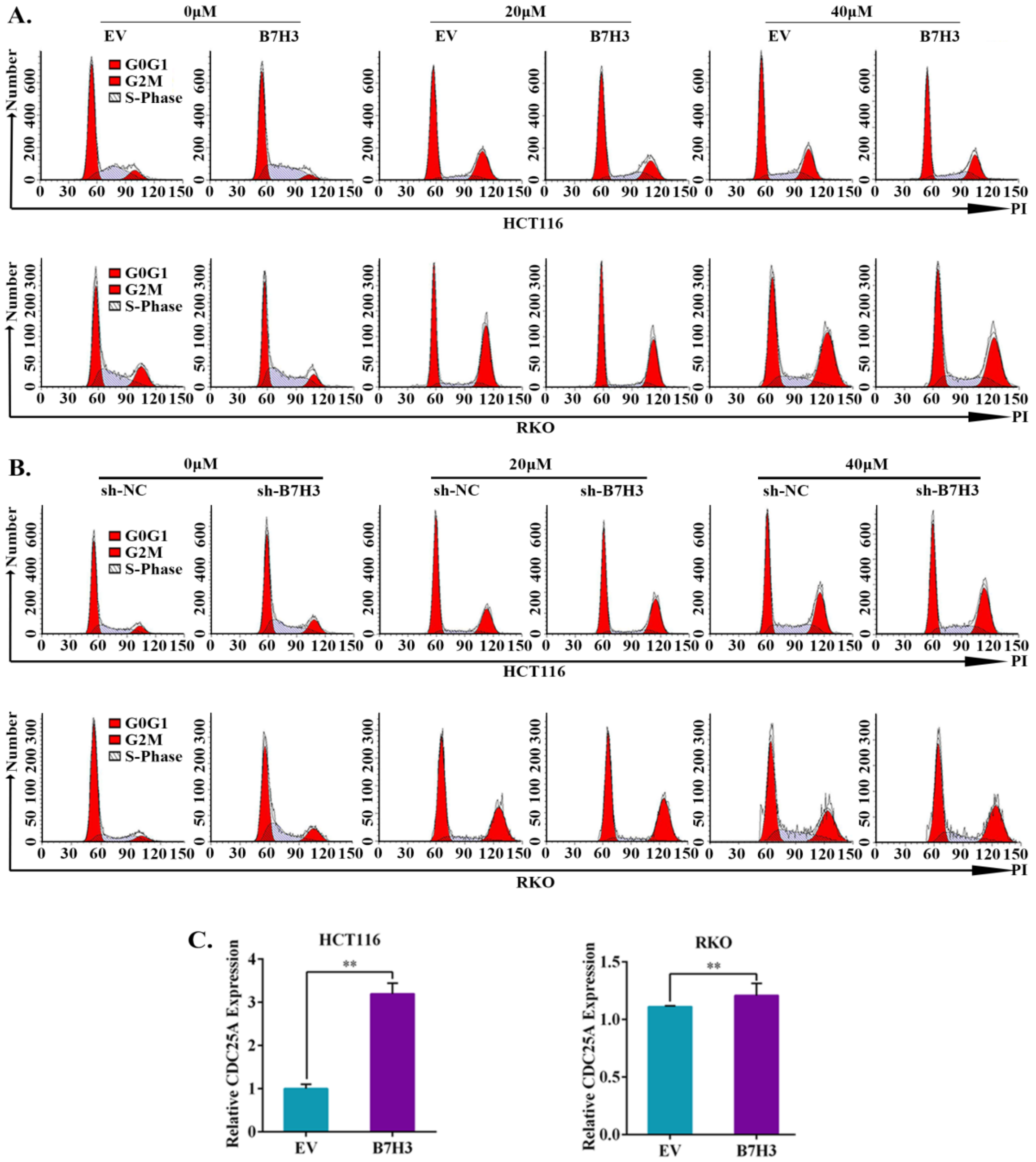
A.
0μM
20μM
40μM
EV
B7H3
G0G1
G2M
S-Phase
Number
PI
HCT116
RKO
B.
sh-NC
sh-B7H3
C.
HCT116
RKO
Relative CDC25A Expression
**
EV
B7H3

**Figure S4**

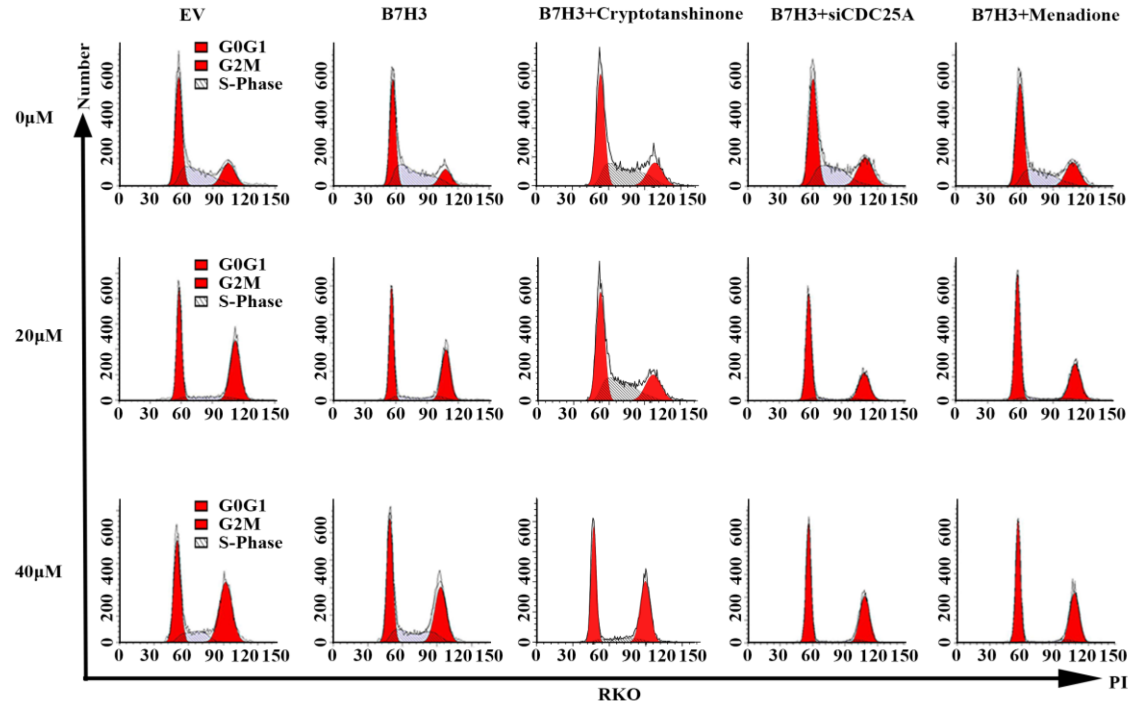
EV
B7H3
B7H3+Cryptotanshinone
B7H3+siCDC25A
B7H3+Menadione
G0G1
G2M
S-Phase
0μM
20μM
40μM
Number
RKO
PI
